# Häufigkeit von Schlafproblemen bei Intensivpflegenden: Eine Post-hoc-Analyse einer Querschnittstudie

**DOI:** 10.1007/s40664-022-00466-w

**Published:** 2022-05-17

**Authors:** Luis Möckel, Ann-Kathrin Hönl, Samantha Gräfe, Florian Jantz, Natalie S. Werner

**Affiliations:** HSD Hochschule Döpfer GmbH, University of Applied Sciences Köln, Waidmarkt 3 & 9, 50676 Köln, Deutschland

**Keywords:** Mentale Gesundheit, Insomnie, Gesundheitsfachberuf, Notfallversorgung, Schichtarbeitende, Mental health, Insomnia, Healthcare professionals, Emergency care, Shift workers

## Abstract

**Hintergrund:**

Intensivpflegende haben durch den Schichtdienst unregelmäßige und ungewöhnliche Arbeitszeiten, welche den natürlichen Hell-Dunkel-Rhythmus stören und somit ein Risiko für Schlafstörungen darstellen können.

**Ziele der Arbeit:**

Ziel dieser Post-hoc-Analyse war es, die Prävalenz selbstberichteter Schlafprobleme sowie damit assoziierte Faktoren bei in Deutschland tätigen Intensivpflegenden zu untersuchen.

**Methoden:**

Hierbei handelte es sich um eine Post-hoc-Analyse der Daten einer Querschnittstudie, welche als Online-Befragung durchgeführt wurde. Es wurden soziodemografische, arbeitsbezogene sowie gesundheitsbezogene Daten erhoben. Mithilfe der Daten wurden die Prävalenz und das dazugehörige 95 %-Konfidenzintervall (95 %-KI) von Schlafproblemen berechnet sowie mittels Chi^2^-Tests, Fisher’s Exact Tests und logistischer Regression Faktoren identifiziert, welche mit diesen assoziiert waren.

**Ergebnisse:**

Der Befragungslink wurde insgesamt 1163-mal angeklickt, und 490 nahmen an der Befragung teil. In die finale Analyse eingeschlossen wurden 432 Intensivpflegende, von denen 82,87 % Frauen waren. Insgesamt berichteten 57,64 % (95 %-KI: 52,83 %; 62,35 %) von Schlafproblemen. Signifikant mit Schlafproblemen assoziiert waren unter anderem das Alter (50 bis 59 Jahre Odds Ratio [OR]: 2,05 [95 %-KI: 1,00; 4,21], *p* = 0,047 vs. 20 bis 29 Jahre) sowie das Leben in einer Familie (OR: 0,50 [95 %-KI: 0,27; 0,93], *p* = 0,029 vs. Single/alleinlebend). Außerdem waren das Depressions- (OR: 1,09 [95 %-KI: 1,06; 1,12], *p* ≤ 0,001), Angst- (OR: 1,10 [95 %-KI: 1,06; 1,14], *p* ≤ 0,001) und Stresslevel (OR: 1,09 [95 %-KI: 1,06; 1,12], *p* ≤ 0,001) signifikant mit Schlafproblemen assoziiert.

**Schlussfolgerung:**

Schlafprobleme lagen bei jeder zweiten teilnehmenden Intensivpflegekraft vor, und insbesondere die mentale Gesundheit war mit Schlafproblemen assoziiert.

## Abstract (en)

**Background:**

Intensive care nurses have irregular and unusual working hours due to shift work, which can disrupt the natural light-dark rhythm and thus increase the risk for sleep disorders.

**Aim of the work:**

The aim of this post hoc analysis was to investigate the prevalence of self-reported sleep problems and associated factors among intensive care nurses working in Germany.

**Methods:**

This analysis was a post hoc analysis of a cross-sectional study, which was performed as an online survey. Collected data were sociodemographic, work-related and health-related data. These data were used to calculate the prevalence and respective 95% confidence intervals (95% CI) of sleep problems. Factors associated with sleep problems were identified using the χ^2^-test, Fisher’s exact test, and logistic regression analysis.

**Results:**

The link to the online survey was accessed 1163 times and 490 intensive care nurses participated in the survey. A total of 432 intensive care nurses were included in this analysis, of whom 82.87% were women. A total of 57.64% (95% CI: 52.83–62.35%) reported sleep problems. Significantly associated with sleep problems were age (50–59 years odds ratio [OR]: 2.05, 95% CI: 1.00–4.21, *p* = 0.047 vs. 20–29 years) and living in a family (OR: 0.50, 95% CI: 0.27–0.93, *p* = 0.029 vs. single/living alone). In addition, depression (OR: 1.09, 95% CI: 1.06–1.12, *p* ≤ 0.001), anxiety (OR: 1.10, 95% CI: 1.06–1.14, *p* ≤ 0.001) and stress (OR: 1.09, 95% CI: 1.06–1.12, *p* ≤ 0.001) levels were significantly associated with sleep problems.

**Conclusion:**

Sleep problems were present in one out of every two participating intensive care nurses and in particular mental health was associated with sleep problems.

Die Versorgung von kritisch kranken Patient*innen auf der Intensivstation erfolgt 24 h an 7 Tagen die Woche. Um diese Gesundheitsversorgung zu gewährleisten, arbeiten viele Pflegekräfte im Schichtdienst. Der Schichtdienst umfasst einen Arbeitsplan, der zwischen den Tagen variieren kann und in der Regel mit Früh‑, Spät- und Nachtdiensten einhergeht [33]. Dadurch werden Pflegekräften ungewöhnliche und unregelmäßige Arbeitszeiten auferlegt, die den zirkadianen Rhythmus stören können. Diese zirkadiane Periodik wird über den Hell-Dunkel-Rhythmus des Tages und die Umwelt synchronisiert. Durch Schichtdienste wird diese Rhythmik beeinträchtigt, indem die Arbeits- und Schlafzeiten vertauscht bzw. verschoben werden. Das kann zur Folge haben, dass in Arbeitsphasen häufig Konzentrationsschwierigkeiten oder Müdigkeit und in Freizeitphasen Schlafprobleme auftreten. Neben dem unnatürlichen Schlaf-Wach-Rhythmus wird durch Schichtdienste auch die Schlafdauer reduziert, da Tageslicht, Lärm und höhere Raumtemperaturen während des Tages häufig den Schlaf stören. Studien zeigen, dass die durchschnittliche Schlafdauer von Menschen im Schichtdienst im Vergleich zu Menschen, die tagsüber arbeiten, verkürzt ist [22, 25]. Menschen im Schichtdienst klagen auch häufiger über Schlafdefizite, Schlafstörungen und Schlafverschiebungen als Menschen im Tagdienst [1, 4, 5]. Tätigkeiten in Schichtarbeit könnten demnach einen wichtigen Risikofaktor für Schlafstörungen darstellen [18].

Schlaf kann in die folgenden Phasen des Non-REM-Schlafes N1 (Einschlafphase), N2 (stabiler Schlaf) und N3 (Tiefschlaf) sowie den REM-Schlaf eingeteilt werden [29]. In der Internationalen statistischen Klassifikation der Krankheiten und verwandter Gesundheitsprobleme [35] werden Schlafstörungen (Insomnien) durch eine ungenügende Schlafdauer und Schlaftiefe gekennzeichnet, die über mindestens 4 Wochen anhält. Dazu zählen Einschlafstörungen, Durchschlafstörungen oder frühmorgendliches Erwachen. Mangelnder Schlaf hat wiederum Auswirkungen auf kognitive, emotionale und soziale Fähigkeit und führt bei den Betroffenen zu einem enormen Leidensdruck, wodurch der Schlaf weiter gestört wird. Dadurch entwickelt sich ein Teufelskreis mit einer Tendenz zur Chronifizierung. Langfristig können eine geringe Schlafdauer und -tiefe die Leistungsfähigkeit und gesundheitliche Lebensqualität mindern. Studien weisen auf ein höheres Risiko für kardiovaskuläre Erkrankungen [30] sowie Übergewicht und Diabetes [2] bei Schlafstörungen hin. In einer Metaanalyse konnte gezeigt werden, dass Schlafstörungen einen Risikofaktor für eine depressive Störung darstellen [3].

Zu erwähnen ist, dass in der Intensivpflege die Folgen von Schlafstörungen nicht nur gesundheitsschädliche Konsequenzen für die Intensivpflegenden selbst, sondern auch für ihre Patient*innen haben können [4, 17, 27]. Es konnte gezeigt werden, dass mit zunehmenden Arbeitsstunden die Pflegequalität abnimmt und mehr Medikationsfehler sowie Beinahe-Unfälle auftreten [27]. Insbesondere bei der komplexen Patientenüberwachung und -kontrolle auf der Intensivstation, bei denen eine künstliche Beatmung und vasoaktive Medikation das Überleben der Patient*innen sichern, können Fehler durch Schlafstörungen schwere Folgen für diese Patient*innen haben [6, 9, 34].

In der ursprünglichen Analyse dieser Studie, welche zur Erfassung von Schmerzen bei Intensivpflegenden durchgeführt wurde, konnten wir zeigen, dass Schlafprobleme signifikant mit wiederkehrenden Schmerzen assoziiert waren [13]. Da die Studie somit zur Ermittlung der Prävalenz von Schmerzen sowie damit assoziierte Faktoren geplant war [13], wurden Daten zur Prävalenz von Schlafproblemen nicht mit analysiert und dargestellt, um eine Vermischung der Hauptdaten mit post hoc analysierten Daten zu vermeiden. Unseres Wissens liegen zum jetzigen Zeitpunkt allerdings keine Daten explizit zur Häufigkeit von Schlafproblemen bei Intensivpflegenden in Deutschland vor. Aus diesem Grund war das Ziel dieser Post-hoc-Analyse, die folgenden Fragestellungen für die Stichprobe aus Intensivpflegenden zu beantworten:

1. Wie hoch ist die Punktprävalenz von selbstberichteten Schlafproblemen jeglicher Art?
2. Wie hoch ist die Punktprävalenz von Schlafproblemen nach Art (Einschlaf‑, Durchschlaf‑, Tiefschlafproblemen)?
3. Welche soziodemografischen, arbeits- und gesundheitsbezogenen Faktoren sind mit Schlafproblemen (jeglicher Art & nach Art) assoziiert?

## Methoden

### Studiendesign und Studienteilnehmende

Bei dieser Arbeit zur Prävalenz von Schlafproblemen handelt es sich um eine Post-hoc-Analyse einer deutschlandweiten Querschnittstudie mit Intensivpflegenden, welche zwischen dem 24.11.2020 und dem 25.01.2021 als Online-Befragung durchgeführt wurde, um die Prävalenz von Schmerzen bei Intensivpflegenden zu untersuchen. Vor der eigentlichen Befragung wurde zunächst ein Pre-Test zwischen dem 17.11.2020 und 23.11.2020 durchgeführt. Teilnehmen konnten alle aktuell in Deutschland als Intensivpflegende tätigen Personen, welche mindestens 18 Jahre alt waren. Die Rekrutierung der Studienteilnehmenden erfolgte im Schneeballprinzip sowie über Online-Gruppen von Intensivpflegenden [13]. Die Befragung wurde mithilfe des Programms SoSci Survey (SoSci Survey GmbH, München, Deutschland) durchgeführt [31]. Es wurde eine Befragung im Online-Format gewählt, um Effekte der sozialen Erwünschtheit möglichst gering zu halten [32]. Weitere Details zum Studiendesign sowie zum Vorgehen sind in Hönl et al. (2021) dargelegt [13].

### Fragebogen

Im Rahmen der Studie wurden mittels selbst entwickelter Fragen unter anderem Daten zu Schmerzen, soziodemografischen und berufsbezogenen Faktoren bei Intensivpflegenden erhoben [13]. Des Weiteren wurde mithilfe des validierten DASS-21 die mentale Gesundheit (Depression, Angst, Stress) der Studienteilnehmenden bewertet [13, 21].

Für die Erhebung der Prävalenz von Schlafproblemen bei Intensivpflegenden wurden ebenfalls selbst entwickelte Items verwendet. So mussten alle Teilnehmenden beantworten, ob diese Schlafprobleme (jeglicher Art) haben (Frage: „*Haben Sie Schlafprobleme?*“ – Antwortmöglichkeiten: Ja/Nein). Wurde das Vorliegen von Schlafproblemen bejaht, so wurden die Studienteilnehmenden gebeten zu spezifizieren, ob es sich um Einschlaf‑, Durchschlaf- oder Tiefschlafprobleme (Frage: „*Welche Art der Schlafprobleme haben Sie an sich festgestellt?*“ – Mehrfachauswahl: Einschlafprobleme, Durchschlafprobleme, Tiefschlafprobleme) handelte, da Personen unter verschiedenen bzw. mehreren Formen von Schlafproblemen leiden können [24]. Einschlafprobleme konnte dabei ein langes Wachliegen von 30 min oder länger vor dem initialen Einschlafen sein. Durchschlafprobleme können beispielsweise ein häufiges wahrgenommenes nächtliches Erwachen mit oder ohne Probleme des erneuten Einschlafens sein. Tiefschlafprobleme haben wir als selbst berichtetes, abnormales nächtliches Verhalten (z. B. Nachtterror, Schlafwandeln) definiert, welches während der N3-Phase des Schlafes auftreten kann [14] und von den Teilnehmenden nicht als Durchschlafprobleme wahrgenommen wurden.

Weitere Angaben zum Studiendesign sowie zum Fragebogen wurden in Hönl et al. (2021) gemacht [13].

### Statistische Auswertung

Es wurden alle Fragebögen in die Analyse eingeschlossen, bei denen 100 % der soziodemografischen und berufsbezogenen Fragen sowie die Frage zum Vorliegen von Schlafproblemen beantwortet wurden. Für die Darstellung der Charakteristika der Studienteilnehmenden wurden die prozentualen Anteile der entsprechenden Gruppen berechnet. Anschließend wurde die Punktprävalenz von Schlafproblemen (jeglicher Art) sowie innerhalb der Population mit Schlafproblemen die Prävalenz der Einschlaf‑, Durchschlaf- und Tiefschlafproblemen berechnet. Zu allen Punktprävalenzen wurden auch die 95 %-Konfidenzintervalle (95 %-KI) bestimmt. Die Prävalenz von Schlafproblemen (jeglicher Art) wurde auch für Subgruppen nach Geschlecht, Altersgruppen, Art der Lebensgemeinschaft, Rauchstatus, Berufserfahrung, Stellenumfang sowie Art des Schichtdienstes berechnet. Für die Einschlaf‑, Tiefschlaf- und Durchschlafprobleme wurde die Berechnung der Prävalenz nach Subgruppen aufgrund der teilweise geringen Anzahl der Teilnehmenden in den einzelnen Subgruppen nicht durchgeführt. Es wurden keine Sensitivitätsanalysen durchgeführt.

Basierend auf den Angaben zu Größe und Gewicht der Teilnehmenden, wurde der BMI berechnet. Der Depressions‑, Angst- und Stressscore der Teilnehmenden wurde ermittelt, indem die entsprechenden Items des DASS-21 aufsummiert und mit dem Faktor 2 multipliziert wurden, um zur Vollversion des DASS äquivalente Werte zu erhalten [11, 13, 19, 20].

Zur Identifikation von mit Schlafproblemen jeglicher Art sowie Einschlaf‑, Durchschlaf- und Tiefschlafproblemen assoziierten Faktoren wurde für kategoriale Variablen (Geschlecht, Altersgruppen, Art der Lebensgemeinschaft, Rauchstatus, Berufserfahrung, Stellenumfang, Schichtdienst) der Chi^2^-Test oder Fisher’s Exact Test und für metrische Variablen (BMI, Depressions‑, Angst‑, Stressscore) die logistische Regression angewendet. Es wurden keine statistischen Methoden zur Kontrolle möglicher Confounding-Faktoren durchgeführt. Des Weiteren wurden Odds Ratios (OR) und dazugehörige 95 %-KI berechnet. Subgruppen von *n* < 10 wurden nicht separat analysiert.

Die statistische Auswertung erfolgte mithilfe des Programms JASP [16].

## Ergebnisse

### Charakteristika der Studienteilnehmenden

Die Befragungslink wurde insgesamt 1163-mal angeklickt, und 490 Intensivpflegende (42,13 % der Klicks) nahmen an der Befragung teil. Die Einschlusskriterien erfüllten insgesamt 432 Teilnehmende (88,16 % aller Befragungen). Die in die vorliegende Post-hoc-Analyse eingeschlossenen Studienteilnehmenden entsprachen der Population, welche bereits in Hönl et al. (2021) hinsichtlich der Prävalenz von Schmerzen analysiert wurde [13].

Insgesamt waren 82,87 % der teilnehmenden Intensivpflegenden Frauen sowie 32,64 % 20 bis 29 Jahre, 32,18 % 30 bis 39 Jahre, 22,92 % 40 bis 49 Jahre, 11,11 % 50 bis 59 Jahre und 1,16 % 60+ Jahre alt (Tab. 1); 63,50 % gaben an, Nichtraucher zu sein, 27,78 % rauchten regelmäßig und 9,72 % gelegentlich. Die Mehrzahl der Studienteilnehmenden (57,64 %) arbeitete in Vollzeit mit einem Stellenumfang von 100 %. Einen Stellenumfang von 75–99 % gaben 26,39 %, einen Umfang von 50–74 % 12,04 % und einen Stellenumfang von < 50 % 3,24 % an. Auf 450 €-Basis arbeiteten 0,69 % der in die Analyse eingeschlossenen Studienteilnehmenden. In Früh‑, Spät- und Nachtschicht arbeiteten 77,79 %, nur in Früh- und Spätschicht 9,95 %, in Früh- oder Spät- oder Nachtschicht 4,63 % und in einem anderen Schichtmodell 7,64 %.

**Table 1:** 

Charakteristika	*N* = 432 Intensivpflegende (in %)
*Geschlecht*	
Frauen	82,87
Männer	17,13
*Altersgruppe*	
20 bis 29 Jahre	32,64
30 bis 39 Jahre	32,18
40 bis 49 Jahre	22,92
50 bis 59 Jahre	11,11
60+ Jahre	1,16
*Rauchstatus*	
Nichtraucher	63,50
Regelmäßige Raucher	27,78
Gelegentliche Raucher	9,72
*Stellenumfang*	
100 %	57,64
75–99 %	26,39
50–74 %	12,04
< 50 %	3,24
450 €-Basis	0,69
*Schichtdienst*	
Früh, spät und Nacht	77,79
Früh und spät	9,95
Früh oder spät oder Nacht	4,63
Andere Variante	7,64

### Prävalenz von Schlafproblemen

**Figure 1:**
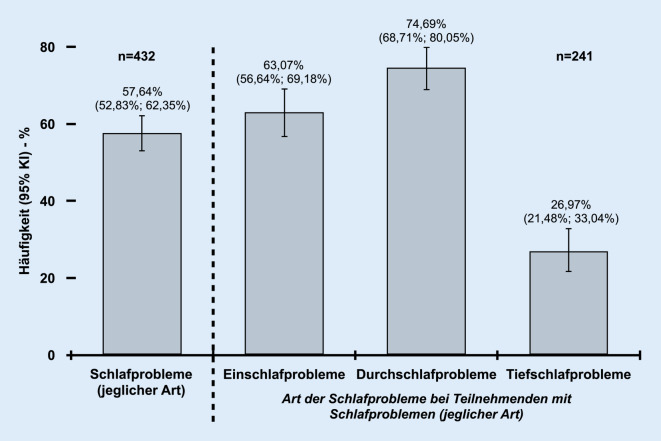

Insgesamt gaben 57,64 % (95 %-KI: 52,83 %; 62,35 %) (249 von 432) teilnehmende Intensivpflegende an, unter Schlafproblemen (jeglicher Art) zu leiden (Abb. 1). Innerhalb der Population mit Schlafproblemen hatten die Durchschlafprobleme mit 74,69 % (95 %-KI: 68,71 %; 80,05 %) (180 von 241) die höchste Prävalenz, gefolgt von den Einschlaf- (63,07 % [95 %-KI: 56,64 %; 69,18 %]; 152 von 241) und den Tiefschlafproblemen (26,97 % [95 %-KI: 21,48 %; 33,04 %]; 65 von 241). Acht Teilnehmende mit Schlafproblemen machten keine weiteren Angaben zu der Art der Probleme.

Bei Betrachtung einzelner Subgruppen konnte die höchste Prävalenz für Schlafprobleme jeglicher Art für die 50- bis 59-Jährigen (72,92 % [95 %-KI: 58,15 %; 84,72 %]), gefolgt von den gelegentlichen Rauchern (64,29 % [95 %-KI: 48,03 %; 78,45 %]) und den Single/alleinlebenden Teilnehmenden (63,21 % [95 %-KI: 53,29 %; 72,37 %]) berechnet werden (Tab. 2). Je nach Schichtmodel zeigten die Teilnehmenden eine Prävalenz der Schlafprobleme (jeglicher Art) von 58,33 % (95 %-KI: 52,86 %; 63,66 %) für diejenigen in Früh‑, Spät- und Nachtschicht, 60,47 % (95 %-KI: 44,41 %; 75,02 %) für Teilnehmende in Früh- und Spätschicht und 60,00 % (95 %-KI: 36,05 %; 80,88 %) für teilnehmende Intensivpflegende, die nur in Früh- oder Spät- oder Nachtschicht arbeiteten. Teilnehmende Intensivpflegende, welche ein anderes als die zuvor genannten Schichtmodelle angaben, zeigten eine Prävalenz für Schlafprobleme von 45,46 % (95 %-KI: 28,11 %; 63,65 %).

**Table 2:** 

Subgruppen	Prävalenz (95 %-KI) Schlafprobleme	Schlafprobleme
		OR (95 %-KI); *p*-Wert
*Geschlecht*		
Frauen (*n* = 358)	58,38 % (53,08 %; 63,54 %)	Referenz
Männer (*n* = 74)	54,05 % (42,07 %; 65,71 %)	0,84 (0,51; 1,39); *p* = 0,493
*Altersgruppe*		
20 bis 29 Jahre (*n* = 141)	56,74 % (48,14 %; 65,05 %)	Referenz
30 bis 39 Jahre (*n* = 139)	53,24 % (44,59 %; 61,74 %)	0,87 (0,54; 1,39); *p* = 0,556
40 bis 49 Jahre (*n* = 99)	56,57 % (46,23 %; 66,50 %)	0,99 (0,59; 1,67); *p* = 0,979
50 bis 59 Jahre (*n* = 48)	72,92 % (58,15 %; 84,72 %)	**2,05 (1,00; 4,21); *p*** **=** **0,047**
60+ Jahre (*n* = 5)	–	–
*Art der Lebensgemeinschaft*		
Single/alleinlebend (*n* = 106)	63,21 % (53,29 %; 72,37 %)	Referenz
In Partnerschaft^a^ (*n* = 138)	63,04 % (54,42 %; 71,10 %)	0,99 (0,59; 1,68); *p* = 0,979
Verheiratet ohne Kinder (*n* = 122)	52,46 % (43,22 %; 61,57 %)	0,64 (0,38; 1,09); *p* = 0,102
Familie^b^ (*n* = 65)	46,15 % (33,79 %; 58,97 %)	**0,50 (0,27; 0,93); *p*** **=** **0,029**
Verwitwet (*n* = 1)	–	–
*Rauchstatus*		
Nichtraucher (*n* = 270)	60,00 % (53,89 %; 65,89 %)	Referenz
Regelmäßige Raucher (*n* = 120)	50,00 % (40,74 %; 59,26 %)	0,67 (0,43; 1,03); *p* = 0,066
Gelegentliche Raucher (*n* = 42)	64,29 % (48,03 %; 78,45 %)	1,20 (0,61; 2,36); *p* = 0,597
*BMI – pro Einheit Anstieg*	–	1,02 (0,99; 1,06); *p* = 0,188
*Depressionsscore – pro Einheit Anstieg*	**–**	**1,09 (1,06; 1,12); *p*** **≤** **0,001**
*Angstscore – pro Einheit Anstieg*	**–**	**1,10 (1,06; 1,14); *p*** **≤** **0,001**
*Stressscore – pro Einheit Anstieg*	**–**	**1,09 (1,06; 1,12); *p*** **≤** **0,001**
*Berufserfahrung*		
1 bis 3 Jahre (*n* = 129)	58,14 % (49,13 %; 66,76 %)	Referenz
4 bis 6 Jahre (*n* = 96)	54,17 % (43,69 %; 64,38 %)	0,85 (0,50; 1,45); *p* = 0,552
7 bis 10 Jahre (*n* = 64)	51,56 % (38,73 %; 64,25 %)	0,77 (0,42; 1,40); *p* = 0,386
11 bis 15 Jahre (*n* = 58)	62,07 % (48,37 %; 74,49 %)	1,18 (0,62; 2,22); *p* = 0,613
15+ Jahre (*n* = 85)	62,35 % (51,18 %; 72,64 %)	1,19 (0,68; 2,09); *p* = 0,538
*Stellenumfang*		
100 % (*n* = 249)	57,83 % (51,43 %; 64,04 %)	Referenz
75–99 % (*n* = 114)	62,28 % (52,72 %; 71,19 %)	1,20 (0,76; 1,90); *p* = 0,423
50–74 % (*n* = 52)	51,92 % (37,63 %; 65,99 %)	0,79 (0,43; 1,43); *p* = 0,434
< 50 % (*n* = 14)	28,57 % (8,39 %; 58,10 %)	**0,29 (0,09; 0,96); *p*** **=** **0,032**
450 €-Basis (*n* = 3)	–	–
*Schichtdienst*		
Früh, spät und Nacht (*n* = 336)	58,33 % (52,86 %; 63,66 %)	Referenz
Früh und spät (*n* = 43)	60,47 % (44,41 %; 75,02 %)	1,09 (0,57; 2,09); *p* = 0,789
Früh oder spät oder Nacht (*n* = 20)	60,00 % (36,05 %; 80,88 %)	1,07 (0,43; 2,69); *p* = 0,883
Andere Variante (*n* = 33)	45,46 % (28,11 %; 63,65 %)	0,60 (0,29; 1,22); *p* = 0,154

^a^Nicht verheiratet und ohne Kinder

^b^Familie definiert als eheliche oder uneheliche Partnerschaft mit mindestens 1 Kind im Haushalt

### Mit Schlafproblemen assoziierte Faktoren

Mit Schlafproblemen (jeglicher Art) (Tab. 2) assoziiert waren das Alter (50 bis 59 Jahre: OR 2,05 [95 %-KI: 1,00; 4,21]; *p* = 0,047 vs. 20 bis 29 Jahre) sowie die Art der Lebensgemeinschaft, wobei in Familien lebende eine signifikant geringere Chance für Schlafprobleme im Vergleich zu Singles/Alleinlebenden aufwiesen (OR: 0,50 [95 %-KI: 0,27; 0,93]; *p* = 0,029). Studienteilnehmende mit einem Stellenumfang von < 50 % zeigten eine signifikant geringere Chance (OR: 0,29 [95 %-KI: 0,09; 0,96]; *p* = 0,032) im Vergleich zu denjenigen mit einer Vollzeitstelle (100 %). Des Weiteren war die mentale Gesundheit, basierend auf den DASS-21-Subskalen, signifikant mit dem Auftreten von Schlafstörungen (jeglicher Art) assoziiert, wobei Anstiege im Depressions- (OR: 1,09 [95 %-KI: 1,06; 1,12]; *p* ≤ 0,001), Angst- (OR: 1,10 [95 %-KI: 1,06; 1,14]; *p* ≤ 0,001) und Stressscore (OR: 1,09 [95 %-KI: 1,06; 1,12]; *p* ≤ 0,001) jeweils die Chance für Schlafprobleme erhöhten. Im Gegensatz dazu zeigten die verschiedenen Formen des Schichtdiensts keine signifikante Assoziation mit dem Auftreten von Schlafproblemen.

Bei Studienteilnehmenden mit Schlafproblemen, war das Auftreten von Einschlafproblemen mit dem Alter, der Art der Lebensgemeinschaft, der Berufserfahrung sowie dem Stellenumfang assoziiert. Für alle gerade genannten Assoziationen wurden ORs von unter < 1 ermittelt, was darauf hindeutet, dass die jeweiligen signifikanten Subgruppen im Vergleich zu den Referenzgruppen eine geringere Chance für das Auftreten von Einschlafproblemen hatten (Tab. 3). Mit Tiefschlafproblemen assoziiert waren ebenfalls das Alter (50 bis 59 Jahre: OR: 0,31 [95 %-KI: 0,11; 0,91]; *p* = 0,027) sowie die mentale Gesundheit (Depressionsscore: OR: 1,05 [95 %-KI: 1,02; 1,08]; *p* = 0,003/Angstscore: OR: 1,05 [95 %-KI: 1,01; 1,08]; *p* = 0,014/Stressscore: OR: 1,05 [95 %-KI: 1,01; 1,09]; *p* = 0,009). Durchschlafprobleme zeigten mit keinem der untersuchten Faktoren eine signifikante Assoziation in der untersuchten Population Intensivpflegender.

**Table 3:** 

Subgruppen	Einschlafprobleme	Durchschlafprobleme	Tiefschlafprobleme
	OR (95 %-KI)	OR (95 %-KI)	OR (95 %-KI)
*Geschlecht*			
Frauen (*n* = 201)	Referenz	Referenz	Referenz
Männer (*n* = 40)	1,11 (0,54; 2,25); *p* = 0,782	1,20 (0,54; 2,69); *p* = 0,654	0,89 (0,41; 1,93); *p* = 0,758
*Altersgruppe*			
20 bis 29 Jahre (*n* = 75)	Referenz	Referenz	Referenz
30 bis 39 Jahre (*n* = 71)	0,66 (0,32; 1,36); *p* = 0,259	1,76 (0,83; 3,71); *p* = 0,138	0,74 (0,37; 1,49); *p* = 0,398
40 bis 49 Jahre (*n* = 56)	**0,39 (0,19; 0,83); *p*** **=** **0,013**	1,73 (0,77; 3,85); *p* = 0,180	0,57 (0,26; 1,24); *p* = 0,156
50 bis 59 Jahre (*n* = 35)	**0,21 (0,09; 0,50); *p*** **≤** **0,001**	1,36 (0,55; 3,34); *p* = 0,503	**0,31 (0,11; 0,91); *p*** **=** **0,027**
60+ Jahre (*n* = 4)	–	–	–
*Art der Lebensgemeinschaft*			
Single/alleinlebend (*n* = 65)	Referenz	Referenz	Referenz
In Partnerschaft^a^ (*n* = 83)	1,17 (0,58; 2,37); *p* = 0,656	1,49 (0,71; 3,15); *p* = 0,293	0,94 (0,45; 1,95); *p* = 0,872
Verheiratet ohne Kinder (*n* = 62)	0,66 (0,32; 1,36); *p* = 0,261	1,09 (0,51; 2,38); *p* = 0,821	0,91 (0,41; 1,99); *p* = 0,810
Familie^b^ (*n* = 30)	**0,36 (0,15; 0,89); *p*** **=** **0,024**	1,36 (0,50; 3,69); *p* = 0,549	1,12 (0,43; 2,90); *p* = 0,817
Verwitwet (*n* = 1)	–	–	–
*Rauchstatus*			
Nichtraucher (*n* = 157)	Referenz	Referenz	Referenz
Regelmäßige Raucher (*n* = 58)	1,57 (0,82; 3,01); *p* = 0,169	0,85 (0,43; 1,71); *p* = 0,654	1,54 (0,80; 2,95); *p* = 0,191
Gelegentliche Raucher (*n* = 26)	1,03 (0,45; 2,45); *p* = 0,921	0,48 (0,20; 1,14); *p* = 0,091	0,70 (0,25; 1,97); *p* = 0,493
*BMI – pro Einheit Anstieg*	0,99 (0,94; 1,03); *p* = 0,516	0,97 (0,92; 1,01); *p* = 0,137	0,99 (0,94; 1,04); *p* = 0,694
*Depressionsscore – pro Einheit Anstieg*	0,99 (0,97; 1,03); *p* = 0,931	1,01 (0,98; 1,04); *p* = 0,594	**1,05 (1,02; 1,08); *p*** **=** **0,003**
*Angstscore – pro Einheit Anstieg*	0,98 (0,95; 1,02); *p* = 0,320	1,01 (0,97; 1,05); *p* = 0,581	**1,05 (1,01; 1,08); *p*** **=** **0,014**
*Stressscore – pro Einheit Anstieg*	1,01 (0,98; 1,05); *p* = 0,445	1,02 (0,98; 1,06); *p* = 0,330	**1,05 (1,01; 1,09); *p*** **=** **0,009**
*Berufserfahrung*			
1 bis 3 Jahre (*n* = 68)	Referenz	Referenz	Referenz
4 bis 6 Jahre (*n* = 51)	0,62 (0,27; 1,44); *p* = 0,267	1,13 (0,50; 2,58); *p* = 0,765	1,31 (0,60; 2,84); *p* = 0,496
7 bis 10 Jahre (*n* = 33)	**0,31 (0,13; 0,77); *p*** **=** **0,010**	1,21 (0,47; 3,15); *p* = 0,694	0,77 (0,30; 1,99); *p* = 0,586
11 bis 15 Jahre (*n* = 36)	**0,29 (0,12; 0,70); *p*** **=** **0,005**	1,16 (0,46; 2,92); *p* = 0,748	0,69 (0,27; 1,76); *p* = 0,432
15+ Jahre (*n* = 53)	**0,23 (0,10; 0,51); *p*** **≤** **0,001**	1,32 (0,58; 3,05); *p* = 0,508	0,63 (0,27; 1,46); *p* = 0,279
*Stellenumfang*			
100 % (*n* = 139)	Referenz	Referenz	Referenz
75–99 % (*n* = 69)	**0,41 (0,22; 0,75); *p*** **=** **0,003**	1,03 (0,53; 1,98); *p* = 0,935	0,88 (0,46; 1,67); *p* = 0,689
50–74 % (*n* = 26)	**0,32 (0,14; 0,76); *p*** **=** **0,008**	2,00 (0,64; 6,18); *p* = 0,224	0,42 (0,14; 1,29); *p* = 0,122
< 50 % (*n* = 4)	–	–	–
450 €-Basis (*n* = 3)	–	–	–
*Schichtdienst*			
Früh, spät und Nacht (*n* = 189)	Referenz	Referenz	Referenz
Früh und spät (*n* = 26)	0,45 (0,20; 1,03); *p* = 0,054	0,95 (0,38; 2,40); *p* = 0,914	0,31 (0,09; 1,07); *p* = 0,053
Früh oder spät oder Nacht (*n* = 12)	1,05 (0,27; 4,94); *p* = 1,00	0,70 (0,18; 3,33); *p* = 0,520	0,79 (0,13; 3,33); *p* = 1,00
Andere Variante (*n* = 14)	0,70 (0,20; 2,56); *p* = 0,567	4,55 (0,58; 35,69); *p* = 0,116	0,65 (0,11; 2,59); *p* = 0,761

^a^Nicht verheiratet und ohne Kinder

^b^Familie definiert als eheliche oder uneheliche Partnerschaft mit mindestens 1 Kind im Haushalt

## Diskussion

Diese Analyse zeigt eine Prävalenz von Schlafproblemen jeglicher Art bei den teilnehmenden Intensivpflegenden von 58 %. Dabei waren Faktoren wie das Alter oder auch die mentale Gesundheit signifikant mit den Schlafproblemen assoziiert, wohingegen die verschiedenen Schichtdienstmodelle keine Assoziation mit Schlafproblemen innerhalb der Studienpopulation hatten.

Schlafstörungen kommen mit einer Prävalenz von 0,05–51 % in westlichen Ländern in der Allgemeinbevölkerung vor [12]. Für die allgemeine deutsche Erwachsenbevölkerung wurde 2013 publiziert, dass je nach Geschlecht etwa 49–53 % unter Einschlaf- und 61–64 % unter Durchschlafstörungen unterschiedlichen Ausmaßes leiden [26]. In der vorliegenden Studie lag die Prävalenz von Schlafproblemen jeglicher Art etwa im Bereich der Allgemeinbevölkerung, wenn auch Einschlaf- und Durchschlafprobleme bei 63 % und 75 % von den Intensivpflegenden mit Schlafproblemen angegeben wurden.

Im Gegensatz dazu berichtete eine chinesische Studie, die ebenfalls während der Coronapandemie durchgeführt wurde, von einer höheren Prävalenz von Schlafstörungen bei Healthcare Professionals im Vergleich zur chinesischen Allgemeinbevölkerung. Allerdings betrug die Prävalenz von Schlafproblemen bei den Healthcare Professionals nur 29,8 % [10]. Eine andere Studie mit Pflegenden zeigte dagegen, basierend auf dem Pittsburgh Sleep Quality Index, eine Prävalenz von Schlafproblemen von 63,9 % [8], welche sicherlich vergleichbar mit der Prävalenz von 58 % für Schlafprobleme (jeglicher Art) bei Intensivpflegenden in der vorliegenden Studie ist.

Eine Studie von Dong et al. (2017) identifizierte bei Pflegenden unter anderem Assoziationen zwischen Schlafproblemen und dem Geschlecht, den Jahren im Beruf, der Häufigkeit von Nachtschichten sowie der Arbeit auf der Intensivstation [8]. Vergleichbar mit den Ergebnissen von Dong et al. (2017) [8], waren die Jahre im Beruf auch bei den teilnehmenden Intensivpflegenden der vorliegenden Studie signifikant mit Einschlafproblemen assoziiert (Tab. 3). Bei den teilnehmenden Intensivpflegenden stellten das Geschlecht sowie die Arbeit in der Nachtschicht allerdings keine mit Schlafproblemen assoziierten Faktoren dar. Dafür waren das Alter (50 bis 59 Jahre) sowie die mentale Gesundheit signifikant mit Schlafproblemen assoziiert (Tab. 2).

Die in dieser Studie nicht vorhandene Assoziation zwischen dem Schichtdienst und Schlafproblemen ist sicherlich als überraschend zu bewerten. Zu erwähnen ist allerdings, dass das Querschnittstudiendesign keine kausalen Zusammenhänge aufzeigen kann und somit ggf. das Studiendesign dieses Ergebnis beeinflusst hat. So wissen wir nicht, ob möglicherweise die Teilnehmenden, welche keine Nachschicht angaben, bereits seit einem längeren Zeitraum unter Schlafproblemen litten und ggf. aufgrund dieser nicht mehr in Nachtschichten arbeiten. Des Weiteren wurde die Studie zwischen dem 24.11.2020 und dem 25.01.2021 durchgeführt und somit in einem Zeitraum, in welchem die bis dato höchste Anzahl intensivmedizinisch behandelter COVID-19-Fälle für Deutschland gemeldet wurde [7]. Es besteht also die Möglichkeit, dass eine hohe Arbeitsbelastung der Intensivpflegenden in diesem Zeitraum auch einen negativen Einfluss auf das Schlafverhalten von Teilnehmenden gehabt haben könnte, welche nicht in der Nachtschicht tätig sind. Dass der Umgang mit COVID-19-Patient*innen einen Effekt zum Beispiel auf die mentale Gesundheit hat, zeigte eine Studie aus Indien. In dieser Studie hatten Healthcare Professionals mit Kontakt zu COVID-19-Patient*innen doppelt so hohe Depressions‑, Angst- und Stresslevels wie Healthcare Professionals ohne direkten Kontakt [15].

Dass es einen Zusammenhang zwischen der mentalen Lebensqualität und Schlafproblemen gibt, wurde bereits durch eine im Jahr 2006 veröffentlichte Studie berichtet [23]. Auch eine signifikante Korrelation zwischen dem Schweregrad der Schlaflosigkeit und dem mentalen Gesundheitsscore wurde bei Notfall- und Rettungskräfte gezeigt [28]. Diese Daten sind somit mit den Ergebnissen der vorliegenden Studie mit Intensivpflegenden vergleichbar (Tab. 2 und 3).

Neben den bereits in Hönl et al. (2021) genannten Limitationen [13] ist für diese Analyse noch zu erwähnen, dass diese ursprünglich nicht geplant wurde, um Faktoren zu identifizieren, die mit Schlafproblemen zusammenhängen. Somit besteht die Möglichkeit, dass Faktoren nicht berücksichtigt wurden, die mit Schlafproblemen assoziiert sind. Des Weiteren waren die Schlafprobleme selbst berichtet, und somit kann die Wahrnehmung, ob Schlafprobleme vorliegen, sehr zwischen den Studienteilnehmenden variieren. Dies trifft insbesondere auf die Tiefschlafprobleme zu. Es sollten somit weitere Studien mit Intensivpflegenden durchgeführt werden, welche explizit für die Thematik geplant sind und validierte Fragebögen zur Erfassung von Schlafproblemen verwenden.

## Fazit für die Praxis

- Diese Post-hoc-Analyse zeigt Schlafprobleme bei über 50 % der Teilnehmenden, wobei die Durchschlafprobleme am häufigsten vorkommen. Des Weiteren konnten einige mit Schlafproblemen assoziierte Faktoren wie höheres Alter und insbesondere die mentale Gesundheit identifiziert werden. In der Praxis könnten somit ältere Intensivpflegende bevorzugt Tagschichten angeboten werden, um den natürlichen Hell-Dunkel-Rhythmus nicht zu stören. Des Weiteren könnte, wenn von den Intensivpflegenden gewünscht, ein regelmäßiges Monitoring der mentalen Gesundheit stattfinden, um besonders belastete Intensivpflegende zu identifizieren und zu entlasten.
- Da die Ergebnisse allerdings nur auf der nachträglichen Analyse von Daten einer Querschnittstudie basieren, sind weitere explizit für diese Thematik geplante und ausführlichere Studien notwendig. Diese zukünftige Forschung sollte, anders als die vorliegende Analyse, mehr bekannte Risikofaktoren für Schlafprobleme beinhalten, aber auch weitere Faktoren aus dem direkten Arbeitsumfeld der Intensivpflegenden. Da wir eine Assoziation mit der mentalen Gesundheit identifizieren konnten, könnte beispielsweise die psychosoziale Arbeitsbelastung der Intensivpflegenden mit untersucht werden. Außerdem sollten Längsschnittstudien durchgeführt werden, welche im Gegensatz zur vorliegenden Querschnittstudie Kausalzusammenhänge identifizieren und somit mögliche Interventionsmöglichkeiten für die Praxis identifizieren könnten.
